# Intravesical explosion during transurethral resection of bladder tumor: a case report and literature review

**DOI:** 10.3389/fonc.2026.1796363

**Published:** 2026-03-19

**Authors:** Hongli Liu, Yao Peng, Zhengsheng Pan, Jianbo Zheng

**Affiliations:** 1 School of Clinical Medicine, Shandong Second Medical University, Weifang, Shandong, China; 2 Department of Urology, Zibo Central Hospital, Zibo, Shandong, China

**Keywords:** bladder rupture, case report, electrosurgery, intravesical explosion, transurethral resection of bladder tumor

## Abstract

Intravesical explosion represents an extremely rare yet hazardous complication during transurethral resection of bladder tumors. Given that severe intravesical explosion can lead to bladder rupture, which requires further surgical intervention and even poses a potential threat to patients’ life safety, this complication deserves heightened clinical attention. Herein, we present an 82-year-old male patient admitted for gross hematuria, dysuria, frequent micturition and urgent micturition lasting over 20 days. Pelvic CT revealed a 43 mm × 30 mm irregular mass on the left side of the bladder dome. He underwent transurethral resection of bladder tumors with warm 0.9% normal saline as irrigation fluid, coagulation power 120 W, resection power 200 W. At 65 minutes into the procedure, a distinct audible “pop” was heard when resecting the tumor base-bladder wall junction, with subsequent tachycardia, heart rate rising from 75 to 110 beats per minute, and failed bladder distension. Laparoscopic exploration confirmed a 50 mm irregular bladder rupture, and radical cystectomy with bilateral cutaneous ureterostomy was performed. Postoperative pathology confirmed invasive sarcomatoid carcinoma of the bladder accompanied by high-grade urothelial carcinoma. The patient recovered uneventfully with no recurrence or metastasis during 9-month follow-up. We further analyze the predisposing factors, therapeutic strategies and preventive measures of this complication by reviewing relevant literature.

## 1 Introduction

Transurethral electrosurgery, including transurethral resection of the prostate (TURP) and transurethral resection of bladder tumors (TURBT), has emerged as a well-established standard surgical modality for the management of benign prostatic hyperplasia and non-muscle-invasive bladder tumors (1, 2). Intravesical explosion represents a rare complication of such procedures, occurring in both TURP and TURBT, and is particularly uncommon in the latter (3). Despite its low incidence, it remains a critical clinical concern due to the considerable potential intraoperative risks it poses. At our institution, a total of 868 TURBT procedures were performed between January 2020 and December 2025, among which one case of bladder rupture secondary to intraoperative intravesical explosion was identified. The patient was diagnosed with invasive sarcomatoid carcinoma of the bladder combined with high-grade urothelial carcinoma, and the simultaneous occurrence of this tumor type and TURBT-related intravesical explosion is rarely reported in clinical practice. Herein, we detail this case and review relevant literature to explore the predisposing factors, therapeutic strategies and preventive measures of this complication, aiming to enrich clinical experience in managing such rare events.

## 2 Case presentation

**Figure 1:**
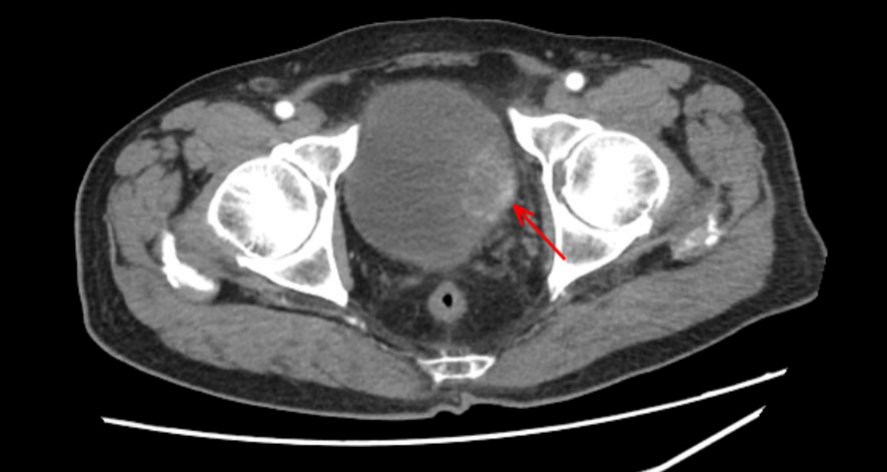
Preoperative CT scan demonstrating a space-occupying lesion within the bladder.

An 82-year-old male patient was admitted to the hospital in February 2025 with a primary complaint of “more than 20 days of gross hematuria, “ accompanied by dysuria, micturition difficulty, frequent micturition and urgent micturition. He denied lower back pain or other symptoms. He had a 10-year history of hypertension, with well-controlled blood pressure under regular medication, no history of diabetes mellitus, coronary heart disease or other comorbidities, and no previous history of abdominal or pelvic surgery. He had no history of neurological disorders, neurogenic bladder, spinal disease, or prior pelvic radiation therapy. The patient did not report chronic urinary retention, long-term catheterization, or previous episodes of acute urinary retention. Preoperative bladder ultrasound revealed a post-void residual urine volume of approximately 51 ml, which was considered mild and did not meet the criteria for chronic urinary retention that could lead to bladder wall thinning. There was no clinical or imaging evidence of significant infravesical obstruction. A pelvic CT scan revealed an irregular 43 mm × 30 mm mass on the left side of the bladder dome with heterogeneous enhancement on dynamic contrast-enhanced scanning (**Figure 1**). No evidence of bladder diverticulum, significant infravesical obstruction, bladder wall thinning, pelvic lymphadenopathy, or distant metastasis was observed. Given the large tumor size and broad-based morphology, clinical judgment indicated that conventional cystoscopic biopsy might fail to obtain representative deep tissue, which could easily lead to deviations in pathological diagnosis and tumor staging. Meanwhile, the patient’s family preferred to attempt comprehensive bladder-sparing treatment. Therefore, after evaluation, the TURBT was planned to clarify the pathological staging and characteristics of the tumor and resect the tumor as completely as possible. Preoperative urinalysis showed an elevated white blood cell count without fever, and anti-infective therapy was administered. After the inflammation was controlled, the TURBT was performed under general anesthesia.

**Figure 2:**
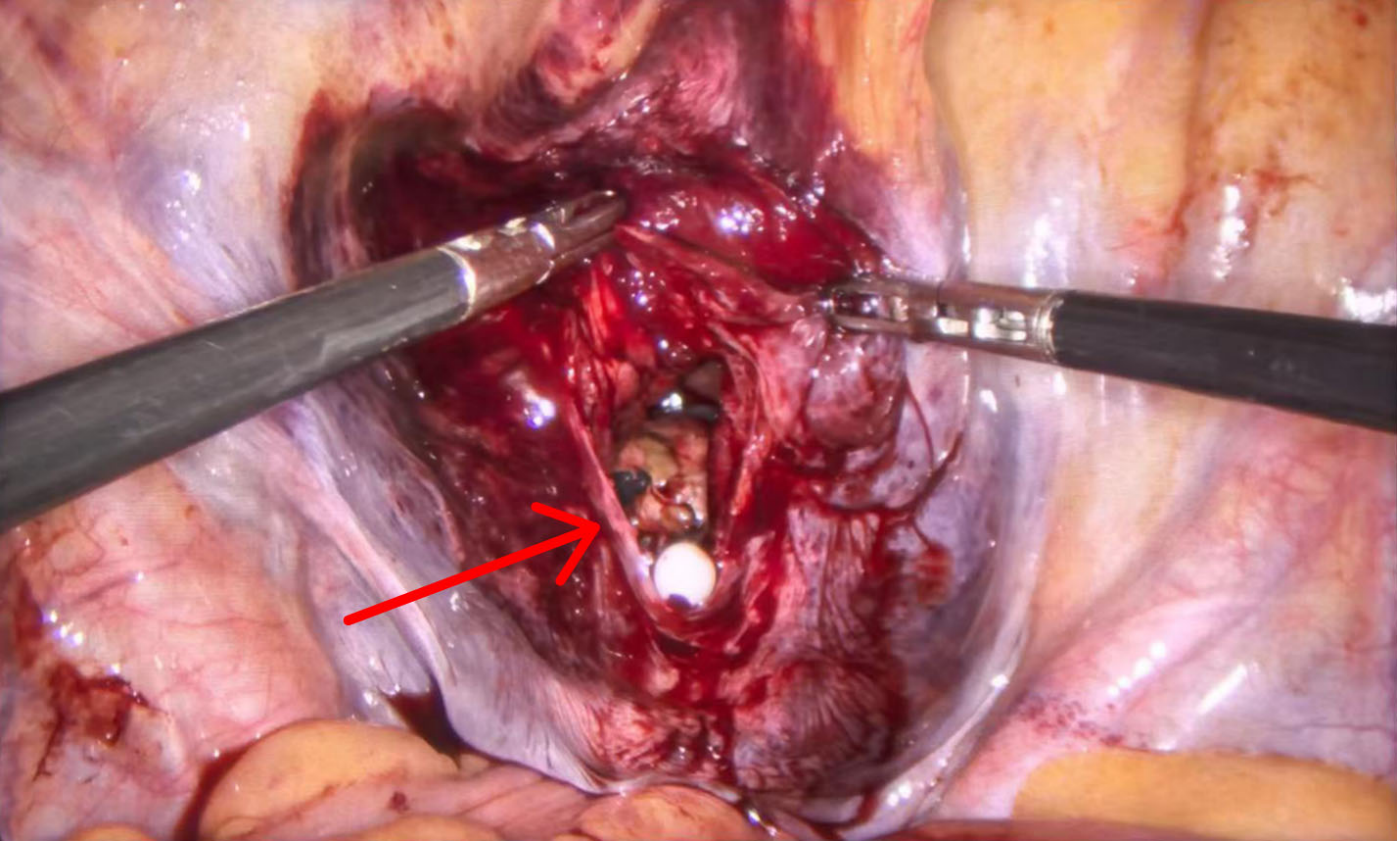
Intraoperative findings revealed an irregular rupture at the bladder dome, with the rupture classified as intraperitoneal. No injury to surrounding organs was observed.

Following induction of general anesthesia, routine disinfection and sterile draping were performed, and a Japanese Olympus F24 plasma resectoscope was inserted under direct vision with the coagulation power set at 120 W and resection power at 200 W; warm 0.9% normal saline was used as the irrigation fluid. Intraoperative findings revealed multiple old blood clots and post-infectious flocculent material in the bladder. After gentle irrigation with an Ellik evacuator, a tumor was identified on the left side of the bladder dome with a flocculent necrotic surface. The tumor was sequentially resected step by step, and the Ellik evacuator was used intermittently to flush out the resected tumor tissue fragments to maintain a clear surgical field. No obturator nerve reflex or sudden thigh adduction was observed during the procedure. At 65 minutes into the procedure, when the resectoscope loop was used to resect the junction between the tumor base on the bladder dome and the bladder wall and was activated under the resection mode at that moment, a distinct audible “pop” was heard. The patient’s heart rate increased rapidly from 75 beats per minute to 110 beats per minute, the bladder could not be distended, the surgical field in the bladder under the resectoscope was bright red, and pale yellow adipose tissue was visible, raising suspicion of bladder rupture. Immediate laparoscopic exploration was performed, revealing an irregular 50 mm rupture on the bladder communicating with the abdominal cavity, with obvious oozing from the wound edges; no injury to the intestinal tract and other abdominal organs was observed (**Figure 2**). Considering the large tumor size, combined with intraoperative bladder rupture leading to abdominal cavity opening accompanied by active bleeding, incomplete tumor resection and an extremely high risk of peritoneal seeding and metastasis of tumor cells, the patient’s condition was fully communicated with the family, and the above risks and pros and cons were explained in detail, including the risks of residual high seeding and postoperative bleeding if perforation repair was attempted, or complete tumor removal via radical surgery. Finally, with the consent of the family, it was decided to perform further radical cystectomy. The bladder rupture was closed with 2-0 barbed sutures. The surgical field was soaked with distilled water for 15 minutes, followed by repeated irrigation with normal saline, and laparoscopic radical cystectomy combined with bilateral cutaneous ureterostomy was routinely performed. Postoperative pathology confirmed exophytic invasive sarcomatoid carcinoma of the bladder accompanied by high-grade urothelial carcinoma, with deep invasion to the deep muscular layer. Surgical margins were negative, and no lymph node metastasis was identified. Approximately 20% of the tumor demonstrated sheet-like necrosis.The patient passed flatus on the second postoperative day, the drainage tube was removed on the fifth postoperative day, and the sutures were removed on the seventh postoperative day, after which the patient was discharged from the hospital. The patient was followed up with reexaminations every 3 months; within 9 months after surgery, the patient’s general condition was good, no special discomfort was reported, and no tumor recurrence or peritoneal seeding and metastasis were observed.

## 3 Discussion

The first documented case of intravesical explosion during transurethral electrosurgery was described by Cassuto in 1926 (4). Intravesical explosion during TURBT results from the accumulation of explosive gases within the bladder, which are then ignited by the high temperatures produced during electrocautery (5). Although rare, several case reports have described this potentially life-threatening complication during TURBT (3, 6). Ning et al. (7) reported through *in vitro* experiments that the gases produced by electrocautery or electrocoagulation of tissue consist primarily of hydrogen (40%-50%), with a small proportion of oxygen. Similarly, Davis et al. (8) reported in their *in vitro* studies that the high temperatures generated during electrocautery-induced tissue electrocoagulation cause the hydrolysis of water within tissue cells, leading to the production of substantial amounts of hydrogen (at least 30%) and minimal oxygen (up to 3%). While hydrogen is highly flammable, the limited amount of oxygen generated during electrocautery or electrocoagulation is insufficient to ignite an explosion. The oxygen required for an explosion typically enters the bladder during the surgical procedure, often owing to poor sealing of the endoscope sheath or to oxygen generated during irrigation. An explosion can only occur when the ratio of combustible gases to oxygen in the bladder reaches a specific threshold and is exposed to high-temperature sparks from electrocautery or electrocoagulation (9, 10). For example, when hydrogen makes up 40% of the volume during electrocautery, the air-hydrogen ratio necessary for an explosion ranges from 11.4% to 90.6%. Without this specific ratio, an explosion cannot occur (11, 12).

Zhang et al. (13) suggested that poor sealing integrity of surgical instruments can facilitate air entry into the bladder, significantly elevating the risk of intravesical explosion. Additionally, when using the Ellik evacuator, it is crucial to fill the container with irrigation fluid without residual air pockets to prevent air from entering the bladder and causing intravesical explosion. Viville et al. (9) reported that continuous irrigation may pose a greater risk than intermittent irrigation, inducing slow and continuous gas accumulation at the bladder apex and triggering intravesical explosion. Erckert et al. (14) conducted a study involving 910 bladder cancer patients and reported that bladder cancer can develop in any part of the bladder, with a notably higher incidence in the trigone and posterior wall. Tumor resection in these regions typically avoids gas accumulation near the bladder dome and anterior wall, thereby reducing the risk of intravesical explosion. Bladder anterior wall and dome tumors are more prone to triggering intravesical explosion due to gas bubble interference but account for a relatively small percentage of bladder cancers, especially those in the anterior wall. In contrast, during prostate surgery, the relatively fixed position of the prostate increases the likelihood of contact with gas accumulated at the top of the prostate fossa and bladder anterior wall, increasing the risk of intravesical explosion during TURP. Large tumor volume is also a risk factor for intravesical explosion, as it prolongs surgical duration, thereby facilitating progressive intravesical gas accumulation, and increases the volume of tissue debris produced during resection; the increased debris load elevates the risk of air entrainment during repeated irrigation with the Ellik evacuator, ultimately inducing intravesical explosion. Most scholars argue that the nature of the irrigation solution is not the primary cause of intravesical explosion. Intravesical explosion has been reported in both TURBT and TURP, regardless of whether the irrigation solution was glycine, mannitol, or distilled water (5, 8, 10). Vincent (15) identified bladder diverticulum as an additional risk factor for intravesical explosion during transurethral resection. Furthermore, neither spinal anesthesia nor intravenous general anesthesia is associated with an elevated risk of intravesical explosion. However, Hirai et al. (16) reported a case of intravesical explosion induced by nitrous oxide inhalation induction anesthesia; nitrous oxide can diffuse into the bladder with blood flow and accumulate in the closed space during surgery, and its flammability is easily ignited by electrocision sparks, thus inhalation anesthesia should be avoided during Transurethral electrosurgery.

Building on the aforementioned potential risk factors, an analysis of the causes underlying this case of intravesical explosion is as follows: First, large bladder tumor volume necessitates a prolonged transurethral resection, resulting in the accumulation of excessive combustible gas. Second, negligent irrigation operation leads to the unintentional entrainment of excessive air into the bladder. Third, overly high power parameters during resection and electrocoagulation generate high-temperature sparks. Notably, the rupture occurred abruptly immediately after the audible explosive sound, rather than progressively during deep resection. The sudden hemodynamic changes and immediate loss of bladder distension further support that the rupture was secondary to an explosive event rather than mechanical perforation caused by excessive resection depth. Although surface necrosis of the tumor was observed intraoperatively, the rupture site was located at the tumor base–bladder wall junction and coincided precisely with the explosive event. Therefore, tumor necrosis alone was unlikely to be the primary cause of bladder rupture in this case.

Bladder injuries occurring during transurethral bladder surgery can range from minor mucosal tears to complete bladder rupture. Large contemporary clinical studies have demonstrated that bladder perforation remains one of the most significant complications of TURBT, with risk factors including a history of previous transurethral resection of bladder tumors, obturator reflex, and a history of intravesical Bacillus Calmette-Guérin (BCG) therapy (17, 18). These findings underscore the importance of careful intraoperative technique, appropriate patient selection, and heightened vigilance, particularly in high-risk cases. The intraoperative diagnosis of bladder rupture relies on several key indicators: an audible explosive sound, abdominal distension on physical examination, an inability to fill the bladder with instilled fluid, and progressive significant changes in vital signs including heart rate, blood pressure, and oxygen saturation (13). Immediate cessation of electrosurgical resection or electrocoagulation is crucial. Vital signs and lower abdominal status should be closely monitored. A resectoscope should be used to carefully observe bladder filling and the integrity of the bladder mucosa (19). In some cases, no obvious injuries may be identified on resectoscopic examination following bladder rupture. In such instances, cystography and other diagnostic modalities may be used to assist in diagnosis to determine the location, severity, and extent of the rupture (20, 21). If the bladder shows no significant injury, the procedure may be resumed after evacuating residual gas and irrigation fluid from the bladder (19). For minor bladder lacerations with scant bleeding, conservative management is warranted; this may include hemostasis under the resectoscope, indwelling catheterization, or suprapubic bladder puncture. Importantly, while suprapubic bladder puncture can be used to mitigate intravesical explosion risk during TURP, it is contraindicated during TURBT because of the risk of bladder tumor cell seeding (19). In cases of severe bladder rupture with persistent active bleeding, immediate laparoscopic repair or open surgery is indicated. Concurrently, abdominal visceral injury should be ruled out, and electrolyte disturbances should be corrected as severe electrolyte derangements can be life-threatening (22, 23). Prior to surgical repair, abdominal paracentesis for aspiration of extravasated irrigation fluid may help prevent or manage electrolyte imbalances (19). Georgios et al. (3) suggested that laparoscopic repair minimizes surgical trauma and facilitates easier aspiration of abdominal fluids and comprehensive assessment of abdominal visceral injuries. However, for patients with large, irregular, or multiple bladder tears, open surgery may be more advantageous instead.

This report has several limitations. First, as a single-case report, the findings cannot be generalized, and definitive conclusions regarding risk stratification or causality cannot be established. Second, the follow-up duration of 9 months is relatively short for assessing long-term oncological outcomes. Although no recurrence or metastasis has been observed thus far, extended follow-up is necessary to determine the patient’s long-term prognosis. Future larger studies are warranted to better define the incidence, risk factors, and preventive strategies of intravesical explosion during TURBT.

The following recommendations are provided to prevent bladder rupture during transurethral bladder surgery. First, for patients with large tumor volumes who are expected to undergo prolonged surgeries, the procedure should be performed by experienced surgeons. This can minimize the surgical duration and reduce the risk of air entering the bladder. Second, for patients with prostate tumors, preoperative suprapubic cystostomy may assist in evacuating gas from the bladder. However, this procedure is contraindicated for bladder tumor patients because of the potential risk of tumor cell dissemination. Third, it is crucial to ensure that all surgical instrument connections are tightly sealed to prevent air ingress. Fourth, gas from the bladder should be promptly evacuated by applying gentle pressure to the lower abdomen while adjusting the angle of the resectoscope sheath. Alternatively, a ureteral catheter can be passed through the resectoscope sheath and placed into the bladder to aspirate gas bubbles (19, 24). Fifth, when filling the Ellik irrigator with saline solution, ensure that all air bubbles are completely expelled. During irrigation, the bottle should be maintained in an upside-down position to prevent air from entering the bladder. The number of irrigations should be minimized to avoid the continuous accumulation of gas in the bladder due to repeated flushing. After each Ellik irrigation, any residual gas should be expelled from the bladder. Sixth, electrocoagulation or electrosurgical resection should be performed using low or medium power settings, and the “touch-and-release” technique with repeated short applications should be employed to avoid the generation of sparks. Seventh, adjusting the surgical position or tilt angle of the operating table can help move gas bubbles away from the surgical field, thus preventing contact between electrosurgical sparks and gas bubbles.

## 4 Conclusion

Severe intravesical explosion can cause bladder rupture and even pose a life-threatening risk to patients, which therefore warrants due clinical attention. Intraoperatively, the ingress of external air into the bladder should be strictly avoided, and if the surgical duration is prolonged, care should be taken to repeatedly empty the bladder of gas to prevent the occurrence of such complications.

## Data Availability

The original contributions presented in the study are included in the article/supplementary material. Further inquiries can be directed to the corresponding author.
